# Transfer RNA Modification Enzymes with a Thiouridine Synthetase, Methyltransferase and Pseudouridine Synthase (THUMP) Domain and the Nucleosides They Produce in tRNA

**DOI:** 10.3390/genes14020382

**Published:** 2023-01-31

**Authors:** Hiroyuki Hori

**Affiliations:** Department of Materials Science and Biotechnology, Graduate School of Science and Engineering, Ehime University, Matsuyama 790-8577, Japan; hori.hiroyuki.my@ehime-u.ac.jp; Tel.: +81-(89)-9278548

**Keywords:** tRNA, tRNA modification enzyme, 4-thiouridine, deaminase, C to U editing, tRNA methyltransferase, N2-methylguanosine, N4-acetylcytidine, pseudouridine synthase, PUS10

## Abstract

The existence of the thiouridine synthetase, methyltransferase and pseudouridine synthase (THUMP) domain was originally predicted by a bioinformatic study. Since the prediction of the THUMP domain more than two decades ago, many tRNA modification enzymes containing the THUMP domain have been identified. According to their enzymatic activity, THUMP-related tRNA modification enzymes can be classified into five types, namely 4-thiouridine synthetase, deaminase, methyltransferase, a partner protein of acetyltransferase and pseudouridine synthase. In this review, I focus on the functions and structures of these tRNA modification enzymes and the modified nucleosides they produce. Biochemical, biophysical and structural studies of tRNA 4-thiouridine synthetase, tRNA methyltransferases and tRNA deaminase have established the concept that the THUMP domain captures the 3′-end of RNA (in the case of tRNA, the CCA-terminus). However, in some cases, this concept is not simply applicable given the modification patterns observed in tRNA. Furthermore, THUMP-related proteins are involved in the maturation of other RNAs as well as tRNA. Moreover, the modified nucleosides, which are produced by the THUMP-related tRNA modification enzymes, are involved in numerous biological phenomena, and the defects of genes for human THUMP-related proteins are implicated in genetic diseases. In this review, these biological phenomena are also introduced.

## 1. Introduction

To date, more than 150 modified nucleosides have been found in RNAs from the three domains of life [1]. Transfer RNA contains numerous modified nucleosides [2,3] and the majority of modified nucleosides in tRNA are introduced by site-specific tRNA modification enzymes. Transfer RNA modification enzymes frequently contain one or more distinct domains in addition to the catalytic domain, although small tRNA methyltransferases such as TrmL [4,5] and TrmH [6,7] are mainly composed of the catalytic domain [8,9,10]. The existence of the thiouridine synthetase, methyltransferases and pseudouridine synthase (THUMP) domain was originally predicted in a bioinformatic study [11]. In this study in 2001, Aravind and Koonin reported that tRNA 4-thiouridine synthetase-like proteins, conserved RNA methyltransferases, archaeal pseudouridine synthases and several uncharacterized proteins share a predicted RNA binding domain, which adopts an α/β fold [11]. At that time, although the *Escherichia coli thiI* gene product had already been identified as a tRNA 4-thiouridine synthetase [12], functions of the other proteins were unknown. Furthermore, no structures for any of the proteins, including ThiI, had been reported. In 2004, the *Pyrococcus abyssi* PAB1283 protein was firstly identified as a tRNA methyltransferase, which contains a THUMP domain [13]. Because the PAB1283 protein possesses enzymatic activity for the formation of *N*^2^-methylguanosine (m^2^G) and *N*^2^, *N*^2^-dimethylguanosine (m^2^_2_G) at position 10 in tRNA, nowadays, the PAB1283 protein is called archaeal Trm11 (arcTrm11). At the same time, the *Saccharomyces cerevisiae tan1* gene product was found to be an essential protein for the formation of *N*^4^-acetylcytidine at position 12 (ac^4^C12) in tRNA^Leu^ and tRNA^Ser^ [14]. Although Tan1 contains a THUMP domain, this protein itself does not possess tRNA acetyltransferase activity [14] and does not contain a catalytic domain [15]. Later, Tan1 was identified as a partner protein of *S. cerevisiae* tRNA acetyltransferse (Kre33) [16]. Since the prediction of the THUMP domain more than two decades ago, many tRNA modification enzymes containing a THUMP domain have been identified. Among them, in addition to tRNA 4-thiouridine synthetases, tRNA methyltransferases, tRNA pseudoridine synthases, tRNA deaminase [17] and a partner protein of tRNA acetyltransferases [16] have been identified. In this review, I focus on functions and structures of these tRNA modification enzymes and the modified nucleosides they produce. Several THUMP-related proteins are involved in not only tRNA modification but also modifications of other RNAs such as rRNA [16,18,19]. In these cases, appropriate reviews and representative articles are introduced due to the limitation of space in this review.

## 2. Classification of THUMP-Related tRNA Modification Enzymes

**Figure 1:**
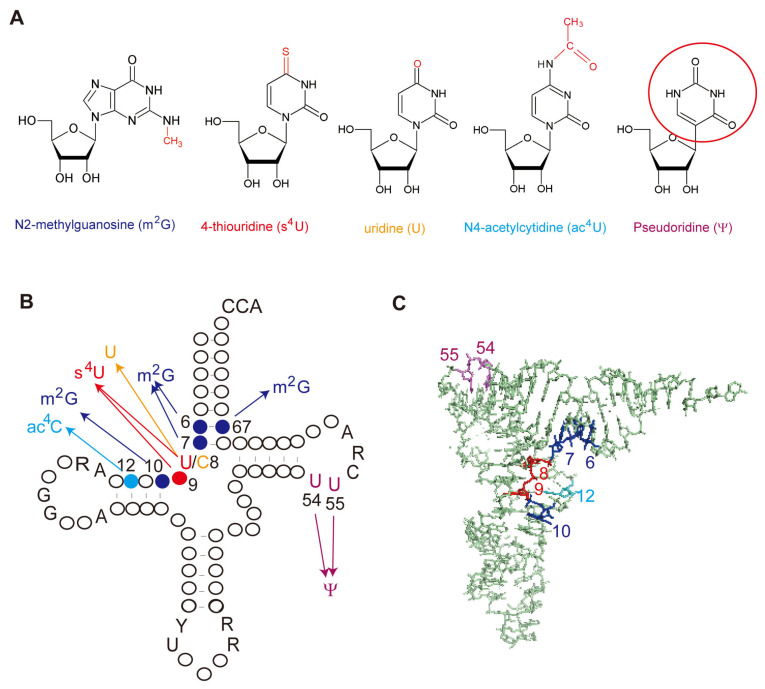
Structures of modified nucleosides, which are produced by THUMP-related tRNA modification enzymes, and their positions in tRNA. (**A**) Structures of modified nucleosides, which are produced by THUMP-related tRNA modification enzymes. Modifications are indicated in red. Because uridine is produced by deamination of cytidine, the 4-*O* atom is colored in red. Because pseudouridine is synthesized by isomerization of uridine, the uracil base is enclosed in a red circle. (**B**) The typical tRNA structure is represented as a cloverleaf model. The numbers show the positions in tRNA. Conserved residues in tRNA are shown as letters: abbreviations, R, purine; Y, pyrimidine. Position 8 is conserved as U (red) in almost all tRNAs; however, in the case of *M. kandleri*, position 8 in precursor tRNA is C (orange). The colors correspond to the modified nucleosides in **A**: blue, m^2^G (and m^2^_2_G); red, s^4^U; orange, U; cyan, ac^4^C; purple, Ψ. *T. kodakarensis* NAT10 homolog acetylates multiple positions in tRNA as described in the main text. (**C**) The modification positions are mapped on the L-shaped yeast tRNA^Phe^ structure.

According to enzymatic activity, THUMP-related tRNA modification enzymes can be classified into five types: 4-thiouridine synthetase, deaminase, methyltransferase, a partner protein of acetyltransferase and pseudouridine synthase (Table 1). As described in a later section, although the classification of tRNA (m^2^G/m^2^_2_G) methyltransferases is complicated, archaeal and eukaryotic Trm11-Trm112 are combined in one column in Table 1. The modification positions and structures of modified nucleosides, which are produced by THUMP-related tRNA modification enzymes, are summarized in Figure 1. TkTHUMDP1-TkNAT10 modify multiple positions in tRNA. For example, when *T. kodakarensis* cells were cultured at 95 °C, C12, C35 and C56 in tRNA^Leu^ were modified to ac^4^C12, ac^4^C35 and ac^4^C56, respectively, by TkTHUMDP1-TkNAT10 [18]. The crystal structure of human PUS10 [20] and a structural model of archaeal Pus10 [21] show that the THUMP domain-related structure is contained in the *N*-terminal accessory domain. The accessory domain is considerably larger than the THUMP domain in other THUMP-related tRNA modification enzymes.

**Figure 2:**
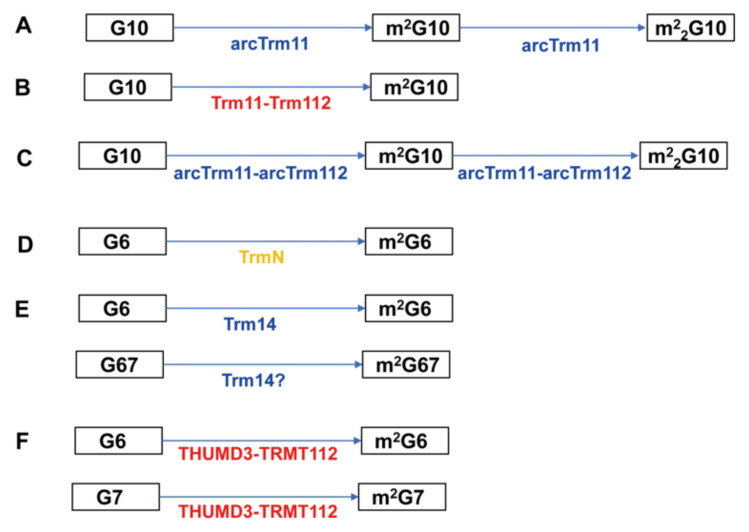
The modification pathways of THUMP-related tRNA methyltransferases. Eukaryotic, archaeal and bacterial enzymes are colored in red, blue and orange, respectively. The modification sites and modified nucleosides are enclosed by squares. (**A**) ArcTrm11 from *P. abyssi* and *T. kodakarensis* produces m^2^G10 and m^2^_2_G10. The m^2^_2_G10 modification is produced by the second methylation from m^2^G10. (**B**) *S. cerevisiae* Trm11 required a partner protein (Trm112) for the methylation and produces only m^2^G10. (**C**) ArcTrm11 from *A. fulgidus* and *Halloferax volcanii* requires a partner protein (arcTrm112) and produces both m^2^G10 and m^2^_2_G10. (**D**) TrmN produces m^2^G6 from G6. (**E**) Trm14 produces m^2^G6 from G6. “?” means that *T. kodakarensis* Trm14 may produce m^2^G67 as well as m^2^G6; this modification has not been confirmed by purified protein. (**F**) Human THUMP3-TRMT112 complex produces m^2^G6 and m^2^G7 from G6 and G7, respectively.

The biosynthesis pathways of modified nucleosides by THUMP-related tRNA methyltransferases are summarized in Figure 2.

## 3. Effect of the Modified Nucleosides, Which Are Produced by THUMP-Related tRNA Modification Enzymes, on tRNA Structure

All modified nucleosides, which are produced by THUMP-related tRNA modification enzymes, are considered to stabilize the L-shaped tRNA structure.

### 3.1. s^4^U8 and s^4^U9

The sulfur atom in s^4^U strengthens the hydrophobic interaction. The melting temperature of tRNA^Ser^ from an *E. coli thiI* gene deletion strain decreases by 4.7 °C as compared to that from the wild-type strain [32]. Therefore, at least s^4^U8 stabilizes the L-shaped tRNA structure. The effect of s^4^U9 modification on the tRNA structure is unknown.

### 3.2. U8

U8 is a conserved nucleoside in tRNA and forms a reverse Hoogsteen tertiary base pair with A14 [33]. Therefore, deamination from C8 to U8 is essential for maintenance of the L-shaped tRNA structure [17].

#### 3.2.1. m^2^G10 and m^2^_2_G10

The m^2^G modification does not disturb the formation of the Watson–Crick base pair with C. The O6 atom of m^2^G10 in the m^2^G10-C25 base pair forms a hydrogen bond with the amino group of G45 in *S. cerevisiae* tRNA^Phe^. Furthermore, the m^2^G10-C25 base pair stacks with the m^2^_2_G26-A44 tertiary base pair. The methyl group in m^2^G10 probably stabilizes this stacking effect. In contrast, m^2^_2_G cannot form a Watson–Crick base pair with C. Instead, m^2^_2_G forms a non-Watson–Crick base pair with U, and the m^2^_2_G10-U25 base pair can be observed in *T. kodakakrensis* tRNA^Trp^ [34]. The two methyl groups in m^2^_2_G probably stabilize the stem structure when an m^2^_2_G-U base pair is formed. Furthermore, the m^2^_2_G10 modification prevents the formation of an incorrect Watson–Crick base pair in tRNA [35].

#### 3.2.2. m^2^G6, m^2^G7 and m^2^G67

As described above, the m^2^G modification does not disturb the formation of the Watson–Crick base pair with C. Although the methyl group in these modified nucleosides probably stabilizes the aminoacyl-stem structure in tRNA, the effect has not been confirmed experimentally.

### 3.3. ac^4^C12 and ac^4^C Modifications in Other Positions

The ac^4^C modification tilts the equilibrium of ribose puckering towards the C3′ endo-form [36]. Furthermore, the ac^4^C modification in a stem structure increases the melting temperature of the stem [37]. Therefore, ac^4^C at position12 and other positions probably stabilizes the L-shaped tRNA structure and codon-anticodon interaction.

### 3.4. Ψ54 and Ψ55

The Ψ55 modification is highly conserved in tRNAs from the three domains of life and form a tertiary base pair with G18 in the L-shaped tRNA structure. The presence of Ψ55 enhances the affinity between the T-arm and the D-arm [38]. Although the structural effect of Ψ54 has not been confirmed experimentally, Ψ54 probably forms a tertiary base pair with A58 (or m^1^A58) and the Ψ54-A58 (m^1^A58) base pair stacks with the G53-C61 base pair in the T-stem. Thus, the Ψ54 modification probably stabilizes the tRNA structure.

## 4. Structures and Enzymatic Properties of THUMP-Related tRNA Modification Enzymes

In this section, the structures of THUMP-related tRNA modification enzymes and their enzymatic properties are introduced. As described below, the THUMP domain captures the 3′-end of RNA (in the case of tRNA, the CCA-terminus). This concept is proposed based on structural, biophysical and biochemical studies of ThiI and is extended to studies of other THUMP-related tRNA modification enzymes.

### 4.1. 4-Thiouridine Synthetase (ThiI)

When the existence of the THUMP domain was predicted [11], ThiI was the only identified tRNA modification enzyme in the list of predicted THUMP-related proteins. ThiI is a tRNA s^4^U synthetase [12]. s^4^U is found at positions 8 and 9 in tRNAs from eubacteria and archaea (Figure 1) [1,2,3]. The biosynthesis pathways of s^4^U are different in eubacteria and archaea [39,40,41,42]. In *E. coli*, the sulfur atom in L-cysteine is activated by cysteine desulfrase (IscS) and is then transferred to tRNA by ThiI in the presence of ATP [43,44,45]. Cysteine residues at positions 344 and 456 in *E. coli* ThiI are essential for the reaction and these residues are considered to form a disulfide bond in the catalytic turnover [46,47]. In contrast, the *iscS* gene is not encoded in the majority of archaea genomes [48]. In the case of *Methanococcus maripuludis*, ThiI contains an Fe-S cluster and S^2−^ is used as a sulfur donor instead of L-cysteine [22,48]. However, the Fe-S cluster type *thiI* gene is not present in some archaea genomes and the biosynthesis pathways in these organisms are still unknown [39,48,49]. During the submission of this manuscript, it was reported that *M. maripuldis* and *P. furiosus* ThiI proteins possess a [4Fe-4S] cluster [50]. Furthermore, it has been proposed that these enzymes be renamed TtuI [50].

**Figure 3:**
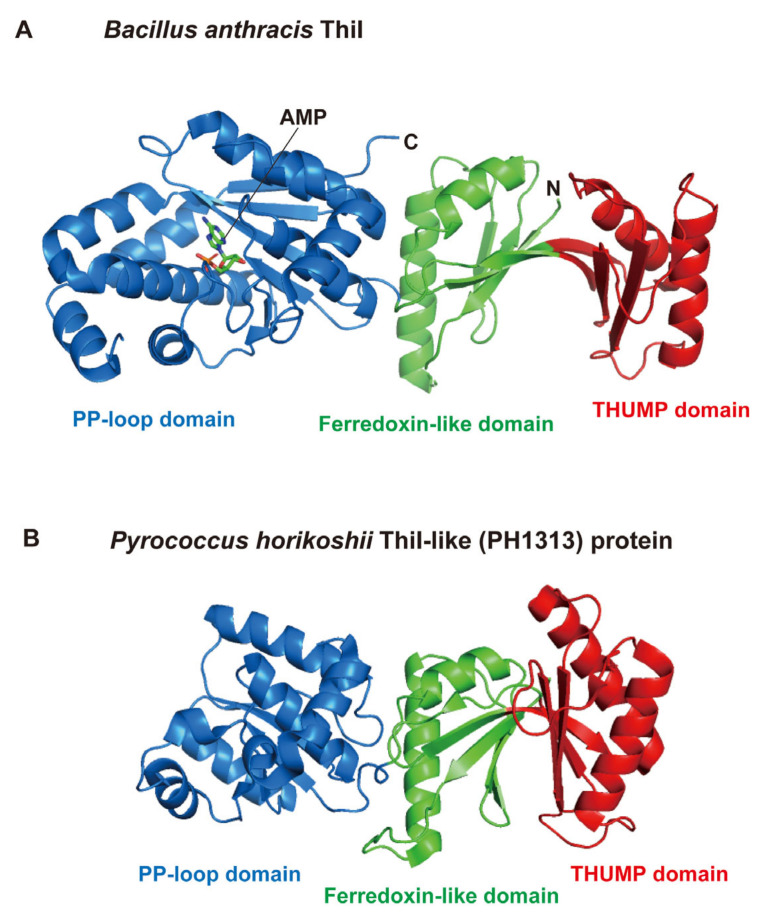
Structures of *B. anthracis* ThiI and *P. horikoshii* ThiI-like (PH1313) protein. (**A**) Structure of *B. anthracis* ThiI (PDB code: 2C5S) is represented by a cartoon model. Ferredoxin-like, THUMP and PP-loop domains are colored in green, red and blue, respectively. *N* and *C* show the *N*- and *C*-termini, respectively. Bound AMP is shown as a stick model. (**B**) Structure of *P. horokoshii* ThiI-like (PH1313) protein (PDB code: 1VBK) is shown by a cartoon model. Although this protein structure was solved as a dimer, only one subunit is shown. Ferredoxin-like, THUMP and PP-loop domains are colored in green, red and blue, respectively. The size of the PP-loop domain of this protein is smaller than that of *B. anthracis* ThiI due to the deletion of the *C*-terminal region.

In 2006, the crystal structure of *Bacillus anthracis* ThiI (PDB code: 2C5S) was the first of the THUMP-related proteins to be reported (Figure 3A) [51]. *B. anthracis* ThiI contains three domains, an *N*-terminal ferredoxin-like domain (green), a THUMP domain (red) and a *C*-terminal PP-loop domain (blue) (Figure 3A). This structure revealed that the THUMP domain is composed of α-helices and β-strands as predicted. A tRNA binding model was also constructed in this study [51]. In the model, the THUMP domain of ThiI was placed near the CCA-terminus of tRNA because it was reported that the CCA-terminus was essential for the sulfur-transfer reaction of ThiI [52]. Later, this idea was experimentally verified by biochemical and structural studies of truncated tRNA [53] and ThiI-truncated tRNA complex [54]. The *N*-terminal ferredoxin-like domain functions to maintain the distance and angle between the THUMP and PP-loop domains. The PP-loop was originally found as a P-loop-like sequence motif, which had been observed in ATP pyrophosphatases [55]. The PP-loop domain in ThiI binds ATP and activates tRNA by adenylation [56,57]. At the same time that the crystal structure of *B. anthracis* ThiI was solved, the structure of *Pyrococcus horikoshii* PH1313 protein (PDB code: 1VBK) was released as a protein of unknown function (Figure 3B) [58]. In the *Pyrococcus* genera, multiple genes for ThiI homologs are often encoded in their genomes [22]. Because ThiI is involved in thiamine biosynthesis in addition to s^4^U modification in tRNA [12,59,60,61], the ThiI homologs in *Pyrococcus* may not have a dual function but instead individual proteins have single roles. Although the structure of the PH1313 protein (Figure 3B) resembles other ThiI proteins, the PH1313 protein lacks several conserved amino acid residues of ThiI proteins. To date, the enzymatic activity of the PH1313 protein has not been confirmed. Furthermore, modified nucleosides in tRNAs from *P. horikoshii* have not been analyzed [62]. Therefore, in this review, the PH1313 protein is described as a ThiI-like protein. The THUMP domain in the *P. horikoshii* ThiI-like protein is also composed of α-helices and β-strands as predicted.

Transfer RNA modification enzymes often recognize local structure(s) in tRNA [63]. Therefore, tRNA modification enzymes are frequently able to modify a truncated tRNA. For example, *E. coli* TrmA [64,65], *E. coli* TruB [66], *E. coli* Tgt [67,68], *T. thermophilus* TrmFO [69], *T. thermophilus* TrmI [70] and *A. aeolicus* TrmD [71] can modify a micro-helix RNA, which mimics the T-arm or anticodon-arm of substrate tRNA. TrmA, TruB, Tgt, TrmFO, TrmI and TrmD are tRNA (m^5^U54) methyltransferase [72], tRNA (Ψ55) synthase [73], tRNA guanine-transglycosylase [67,74,75,76], *N*5, *N*10-methylenetetrahydrofolate-dependent-tRNA (m^5^U54) methyltransferase [77], tRNA (m^1^A58) methyltransferase [78] and tRNA (m^1^G37) methyltransferase [79], respectively. Furthermore, *E. coli* TrmJ [80], *A. aeolicus* TrmB [81] and *T. thermophilus* TrmH [82] can methylate a truncated tRNA. TrmJ, TrmB and TrmH are tRNA (Cm32/Um32) methyltransferase [83], tRNA (m^7^G46) methyltransferase [84] and tRNA (Gm18) methyltransferase [6,85], respectively.

**Figure 4:**
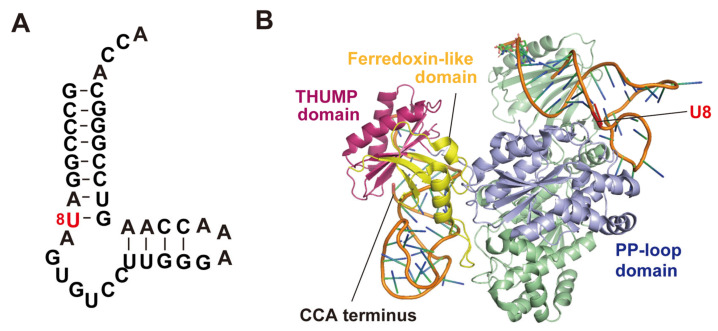
(**A**) Secondary structure of minimum substrate RNA for ThiI. The modification position (U8) is colored in red. This RNA is a truncated RNA of *E. coli* tRNA^Phe^. The secondary structure is based on the complex of minimum substrate RNA and ThiI shown in panel B. (**B**) Crystal structure of the complex of the minimum substrate and *T. maritima* ThiI (PDB code: 4KR6). ThiI forms a dimer structure. To distinguish between the two subunits, one subunit is colored in pale green. The ferredoxin-like, THUMP and PP-loop domains in one subunit are colored in yellow, magenta and pale blue, respectively. The THUMP domain captures the CCA terminus of one minimum substrate RNA. The PP-loop domain in this subunit accesses U8 (red) in another minimum substrate RNA.

Lauhon et al. have reported that a truncated tRNA^Phe^ (Figure 4A) is a minimum substrate for *E. coli* ThiI [52]. This truncated tRNA^Phe^ is also recognized by *Thermotoga maritima* ThiI as a substrate [54]. The crystal structure of the complex of the minimum substrate RNA and *T. maritima* ThiI has been reported (Figure 4B) [54]. *T. maritima* ThiI forms a dimer and two minimum substrate RNAs bind to this dimer. The THUMP domain in one subunit captures the CCA terminus of one minimum substrate RNA and the PP-loop domain in this subunit accesses the modification site (U8) in another minimum substate RNA. Thus, this complex structure demonstrates that ThiI acts as a dimer. The disulfide bond, which acts in the catalytic cycle, in *E. coli* ThiI is formed within a single subunit [86]. Furthermore, this structure proposes a concept that the THUMP domain recognizes the 3′-end of RNA (in the case of tRNA, the CCA terminus).

### 4.2. Deaminase

**Figure 5:**
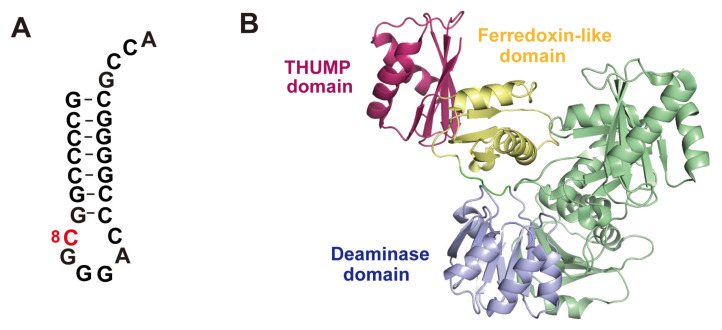
(**A**) Structure of mini-helix RNA. The modification position (C8) is colored in red. (**B**) Crystal structure of CDAT8. CDAT8 forms a dimer structure. To distinguish subunits, one subunit is colored in pale green. Deaminase, ferredoxin-like and THUMP domains in the other subunit are colored in pale blue, yellow and magenta, respectively.

*M. kandleri* is a hyper-thermophilic archaeon in which position 8 in 30 tRNA genes is encoded as C [87,88]. This C8 is modified to U8 by deamination (C to U editing) [17]. For further information about deamination in tRNA, see this review [89]. The enzyme responsible for deamination of C8 is CDAT8. CDAT8 can modify C8 in a micro-helix RNA (Figure 5A). A crystal structure of CDAT8 has been reported (Figure 5B; PDB code, 3G8Q) [17]. The domain arrangement of CDAT8 is different from that of ThiI. From the *N*-terminus to the *C*-terminus, the order of the domains is deaminase, ferredoxin-like and THUMP. However, the structure of the ferredoxin-like and THUMP domains is very similar to that of ThiI. From the model of the complex between CDAT8 and tRNA, it was predicted that the THUMP domain of CDAT8 captures the CCA terminus of substrate tRNA [17].

### 4.3. Methyltransferase

Of the different modified nucleosides in tRNA, methylated nucleosides are the most abundant [1,2,90]. Consistent with this, numerous tRNA methyltransferases have been identified [90]. Transfer RNA methyltransferases can be divided into two types according to the methyl group donor. The majority of tRNA methyltransferases use S-adenosyl-L-methionine as a methyl group donor whereas mnmG (previous name, GidA) [91,92,93,94,95,96] and TrmFO [69,77,97,98] are an exception and use *N*5, *N*10-methylenetetrafolare. S-adenosyl-L-methionine-dependent tRNA methyltransferases are further classified on the basis of their catalytic domain [9,90,99]. The majority of S-adenosyl-L-methionine-dependent tRNA methyltransferases possess a Rossmann fold catalytic domain [9,99]. The second group of S-adenosyl-L-methionine-dependent tRNA methyltransferases belong to a SpoU-TrmD (SPOUT) superfamily, which possess a SPOUT catalytic domain [9,100]. In addition, TrmO is an exception and has a b-barrel type catalytic domain [101].

All THUMP-related tRNA methyltransferases reported possess a Rossmann fold catalytic domain and synthesize only m^2^G (and m^2^_2_G) (Figure 1 and Figure 2 and Table 2). Several enzymes synthesize m^2^_2_G from m^2^G by a second methylation and act on multiple positions (Figure 2). Although classification of tRNA (m^2^G/m^2^_2_G) methyltransferases is complicated, the THUMP-related tRNA (m^2^G/m^2^_2_G) methyltransferases can be divided into two types according to their methylation sites (Table 2). Thus, Trm11/arcTrm11/arcTrm11-arcTrm112/TRMT11-TRMT112 act on position 10 in tRNA, whereas TrmN/Trm14/THUMPD3-TRMT112 act on position 6 and an additional site. It should be mentioned that tRNA (m^2^G/m^2^_2_G) methyltransferases, which do not possess a THUMP domain, do exist. One major group of such tRNA (m^2^G/m^2^_2_G) methyltransferases is the Trm1 family [102,103,104,105,106,107,108,109,110]. *S. cerevisiae* Trm1 catalyzes the methylation of G26 in tRNA and synthesizes m^2^G26 and m^2^_2_G26 [102,103]. Mammalian and *Aquifex aeolicus* Trm1 enzymes form m^2^G27 and m^2^_2_G27 in addition to m^2^G26 and m^2^_2_G26 [105,107]. Crystal structures of *P. horikoshii* [109] and *A. aeolicus* [110] Trm1 proteins demonstrate that these proteins possess a distinct *C*-terminal domain instead of a THUMP domain.

Trm112, TRMT112 and arcTrm112 are hub-proteins (Figure 2 and Table 2), which regulate multiple methyltransferases [23,24,27,111,112,114,115,116]. In the case of human TRMT11-TRMT112, formation of the complex has been reported [111]. However, the modification, position and substrate tRNAs of human TRMT11-TRMT112 have not been experimentally confirmed. For *T. kodakarensis* Trm14, tRNA^Trp^ from a *trm14* gene deletion strain loses the m^2^G67 modification [113]. However, subunit composition and enzymatic activity of *T. kodakarensis* Trm14 have not been confirmed with a purified enzyme. In addition, recently, RNA fragments from tRNA mixtures purified from *M. Jannaschii* [117], *M. maripaldis*, *P. furiosus* and *Sulfolobus acidocaldarius* [118] were analyzed by mass-spectrometry. m^2^G6 and m^2^G67 were observed in several tRNAs from *M. Jannaschii* [117], and thus Trm14 is probably involved in the formation of these modifications. Furthermore, in the case of *P. furiosus*, several tRNAs were shown to possess a m^2^_2_G6 modification in addition to m^2^G6 and m^2^G67 modifications [118]. Therefore, archaeal Trm14 proteins may possess broader positional specificity than was previously thought.

As described in the Introduction, the *P. abyssi* PAB1283 protein (arcTrm11) was the first tRNA methyltransferase identified as containing a THUMP domain [13]. The THUMP domain of *P. abyssi* arcTrm11 has been expressed in *E. coli* cells, purified and analyzed [119]. This study [119] reported that the THUMP domain autonomously folds and that the affinity of the THUMP domain for tRNA is very weak. In 2005, it was reported that *S. cerevisiae* Trm11 requires a partner subunit, Trm112 [23]. Furthermore, the *S. cerevisiae* Trm11-Trm112 complex only produces m^2^G10 in tRNA [23] whereas arcTrm11 produces m^2^G10 and m^2^_2_G10 [13,24,34]. Moreover, in several archaea, arcTrm11 requires arcTrm112 for enzymatic activity as seen with *S. cerevisiae* Trm11 [24,112].

*T. thermophilus* TrmN is the only eubacterial THUMP-related tRNA methyltransferase reported [25]. TrmN methylates G6 in tRNA^Phe^ and produces m^2^G6 [25]. *Methanococcus jannaschii* Trm14 is an archaeal homolog of TrmN and produces m^2^G6 (and m^2^_2_G6) in tRNA^Cys^ [26]. Furthermore, in in vitro experiments, the second methylation from m^2^G6 to m^2^_2_G6 in the tRNA^Cys^ transcript was observed [26]. The human THUMPD3-TRMT112 complex methylates G6 and G7 in several tRNAs and produces m^2^G6 and m^2^G7 [27].

**Figure 6:**
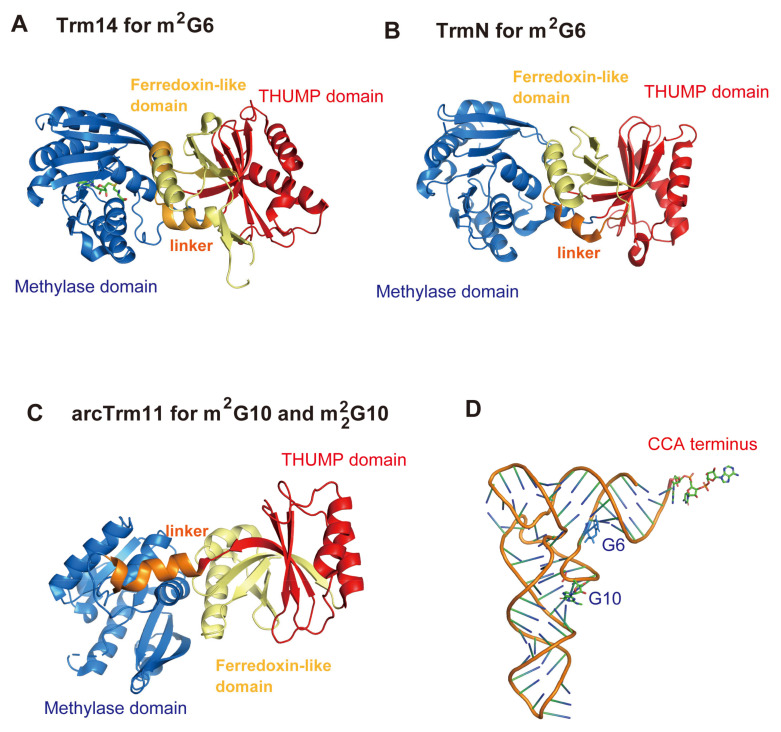
Structures of *P. abyssi* Trm14 ((**A**): PDB code, 3TM4), *T. thermophilus* TrmN ((**B**): PDB code, 3TMA) and *T. kodakarensis* arcTrm11 ((**C**): PDB code, 5E71) are compared. The *N*-terminal ferredoxin-like domain, THUMP domain, Rossmann fold methyltransferase (methylase) domain and linker region are colored in yellow, red, blue and orange, respectively. Trm14 and TrmN modify G6 in tRNA while arcTrm11 modifies G10. The modification sites (G6 and G10) are mapped onto the L-shaped tRNA structure (**D**). G6, G10 and CCA terminus are highlighted as stick models. The distance between the THUMP and methylase domains of Trm14 and TrmN is shorter than that seen in arcTrm11. Because the THUMP domain captures the CCA terminus in tRNA, this short distance between the THUMP and methylase domains of Trm14 and TrmN enables the catalytic pocket in the methylase domain to access the modification site G6. In contrast, the longer distance between the THUMP and methylase domains of arcTrm11 is required for the positioning of the catalytic pocket with respect to the modification site G10. Thus, the *N*-terminal ferredoxin-like domain and linker region are important for the maintenance of the distance and angle between the THUMP and methylase domains, which decides the modification site in tRNA.

In 2012, crystal structures of *P. abyssi* Trm14 (Figure 6A) and *T. thermophilus* TrmN (Figure 6B) were reported [120]. Both enzymes methylate G6 in tRNA and produce m^2^G6. The crystal structures revealed that these enzymes possess a *N*-terminal ferredoxin-like domain, a THUMP domain, a Rossmann fold methyltransferase (methylase) domain and a linker region. In the same study, it was reported that several positively charged amino acid residues are involved in tRNA binding [120]. Furthermore, the structures of the ferredoxin-like domain and the THUMP domain of Trm14 and TrmN are remarkably similar to those of ThiI and CDAT8. In 2016, the crystal structure of *T. kodakarensis* arcTrm11 was solved (Figure 6C) [34]. The arrangement of the domains of arcTrm11 is the same as that of Trm14 and TrmN. However, the distance between the THUMP and methylase domains in arcTrm11 is longer than that in Trm14 and TrmN due to structural differences in the ferredoxin-like domain and the linker region. This difference is important for the selection of the modification site (G10 or G6) (Figure 6D). A site-directed mutagenesis study showed that the THUMP domain in arcTrm11 captures the CCA terminus of substrate tRNA [34]. The distance between the CCA terminus and G10 in tRNA is longer than the distance between the CCA terminus and G6 (Figure 6D). Thus, these crystal structures led to the idea that the methylation site (G6 or G10) is determined by the distance from the THUMP domain to the catalytic pocket.

Eukaryotic and some archaeal Trm11 proteins require a partner subunit (Trm112, TRMT112 or arcTrm112) for enzymatic activity [23,24,27,111,112,114,115,116]. It should be mentioned that eukaryotic Trm112 homologs activate multiple methyltransferases. For example, *S. cerevisiae* Trm112 activates Trm9 [121], Bud23 [122,123] and Mtq2 [124,125] in addition to Trm11. Furthermore, a human ortholog of Trm112, TRMT112 interacts with at least seven human methyltransferases (WBSCR22 (responsible for formation of 7-methylguanosine at position 1636 in 18S rRNA) [126], METTL5 (formation of *N*^6^-methyladenosine at position 1832 in 18S rRNA) [127], HEMK2 (methylation of a glutamine side chain of eRF1 protein) [128], ALKBH8 (responsible for 5-methoxycarbonylmethyluridine derivatives at position 34 in tRNA) [129,130,131,132], TRMT11 [111], THUMPD2 (function unknown) [111] and THUMPD3 (production of m^2^G6 and m^2^G7 in tRNA)) [27].

Several tRNA modification enzymes form protein complexes [90,91,96,116,133,134,135,136]. The partner subunit(s) is frequently involved in the substrate tRNA recognition. Consequently, the binding sites of these modification enzymes are often extended over the whole tRNA molecule. For example, as described in Section 4.1., bacterial tRNA (m^7^G46) methyltransferase (TrmB) can methylate a truncated tRNA, in which the interaction between the T-arm and D-arm is disrupted [81]. However, in contrast, eukaryotic tRNA (m^7^G46) methyltransferase (Trm8-Trm82) [136] requires the interaction between the T-arm and D-arm for methylation [137]. Thus, the existence of Trm82 seems to act on recognition of the L-shaped tRNA structure. In the case of *S. cerevisiae* Trm7, the partner subunits (Trm732 and Trm734) decide the modification positions: Trm7-Trm732 and Trm7-Trm734 catalyze 2′-*O*-methylations at position 32 and position 34, respectively, in tRNA [138]. The biochemical and structural studies of Trm7-Trm734 suggest that Trm734 captures the D-arm in substrate tRNA and controls the accession of the modification site (ribose at position 34) in tRNA to the catalytic pocket in Trm7 [139]. A conserved motif (RRSAGLP sequence) in Trm732 is involved in the methylation of position 32 in tRNA^Phe^ [140]. Thus, the presence of a partner subunit is frequently involved in substrate tRNA recognition.

**Figure 7:**
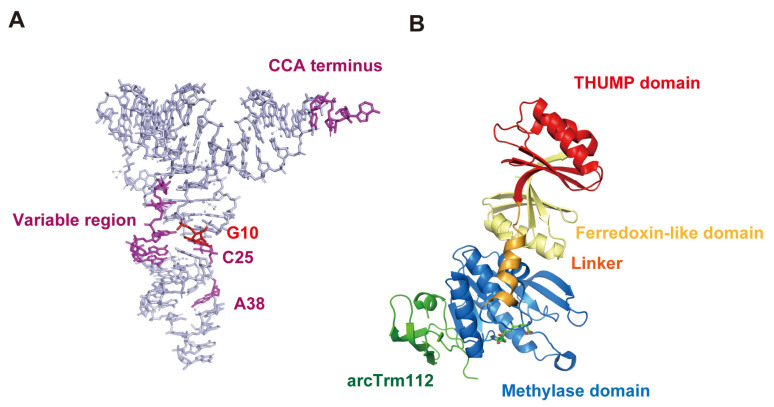
(**A**) Recognition sites of *S. cerevisiae* Trm11-Trm112 are marked on the L-shaped tRNA structure. The modification site (G10) and other recognition sites are colored in red and magenta, respectively. *S. cerevisiae* Trm11-Trm112 methylates standard tRNAs, which possess a regular size (5 nt) variable region, G10-C25 base pair and purine38 in addition to the CCA terminus. (**B**) Crystal structure of *A. fulgidus* arcTrm11-arcTrm112 (PDB code, 6ZXW) is represented by a cartoon model. The ferredoxin-like domain, THUMP domain, Rossmann fold methylase domain, and linker region are colored in yellow, red, blue and orange, respectively. Archaeal Trm112 is colored in green.

*S. cerevisiae* Trm11-Trm112 does not methylate truncated tRNAs [141]. This observation suggests that the binding sites of Trm11-Trm112 in tRNA are spread over the whole tRNA molecule. Biochemical and biophysical studies of *S. cerevisiae* Trm11-Trm112 resulted in the proposal of a model in whichTrm112 is accessible to the anticodon-loop region in tRNA dependent on the movement of the THUMP domain [142]. The required elements in tRNA for methylation by Trm11-Trm112 have been clarified (Figure 7A): the CCA terminus, G10-C25 base pair, regular size (5 nt) variable region and ribose-phosphate backbone around purine38 in tRNA are essential for methylation by *S. cerevisiae* Trm11-Trm112 [141]. Thus, the biochemical study [141] supports the model referenced [142] because the ribose-phosphate backbone around position 38 is recognized by *S. cerevisiae* Trm11-Trm112. Furthermore, the crystal structure of *A. fulgidus* arcTrm11-arcTrm112 has been reported (Figure 7B) [24]. When the THUMP domain in arcTrm11 captures the CCA terminus in substrate tRNA, arcTrm112 accesses the anticodon-loop. Therefore, tRNA recognition mechanisms of eukaryotic and archaeal Trm11-Trm112 seem to be basically common. Human THUMPD3-TRMT112 requires the CCA terminus for methylation and does not methylate a mini-helix RNA [27]. Therefore, TRMT112 in THUMPD3-TRMT112 may also be involved in the anticodon-loop recognition as per Trm11-Trm112.

### 4.4. Acetyltransferase

As described in the Introduction, *S. cerevisiae* Tan1 (human THUMPD1) contains a THUMP domain and acts as a partner protein of tRNA acetyltransferse, Kre33 (human NAT10) [16]. The *Methanothermobacter thermautotrophicus* Tan1 homolog is composed of *N*-terminal ferredoxin-like and *C*-terminal THUMP domains [15]. Although the crystal structure of Kre33 (or NAT10) has not been reported, a structural model (PDB code, 2ZPA) has been proposed [16] in which Kre33 (NAT10) contains DUF1726 (of unknown function), helicase, N-acetyltransferase and tRNA binding domains. In the case of *T. kodakarensis* TkNAT10 (the archaeal homolog of NAT10), the *C*-terminal region is missing [18]. Kre33 catalyzes the acetylation of 18S rRNA as well as acetylation of tRNA [16]. A random mutagenesis study of *T. kodakarensis* revealed that the disruption of the Tk0754 gene causes complete loss of ac^4^C modification in a tRNA mixture [143]. Detailed enzymatic activity of the Tk0754 gene product (TkNAT10) has been reported [18]. In this study, TkNAT10 was shown to modify multiple positions in various RNAs including tRNAs, and the rate of acetylation is increased according to increase in temperature [18]. Yeast two-hybrid system experiments have shown that Tan1 and Kre33 form a complex [16]; however, the structure of the Tan1 and Kre33 complex has not been reported. For details of acetylation of rRNA and other RNAs, see these references [16,18,19].

### 4.5. Pseudouridine Synthase

Pseudouridine (Ψ) is abundant in RNAs from the three domains of life [1,2,3] and is synthesized by C5-ribosyl isomerization from uridine, which is catalyzed by pseudouridine synthases [144,145,146,147,148,149,150]. Pseudouridine synthases can be classified into six families; however, PUS10 is the only THUMP-related enzyme [28,29,144,145,146,147,148,149,150]. In 2006, Ψ55 formation in tRNA catalyzed by archaeal Pus10 was reported [28]. Thus, this report demonstrates that one of the predicted THUMP-containing proteins [11] has pseudouridine synthase activity. In 2008, it was reported that archaeal Pus10 can synthesize Ψ54 in tRNA in addition to Ψ55 [29]. Furthermore, *Methanocaldoccus jannaschii* PUS10 can modify U54 and U55 in a micro-helix RNA, which mimics the T-arm [151].

**Figure 8:**
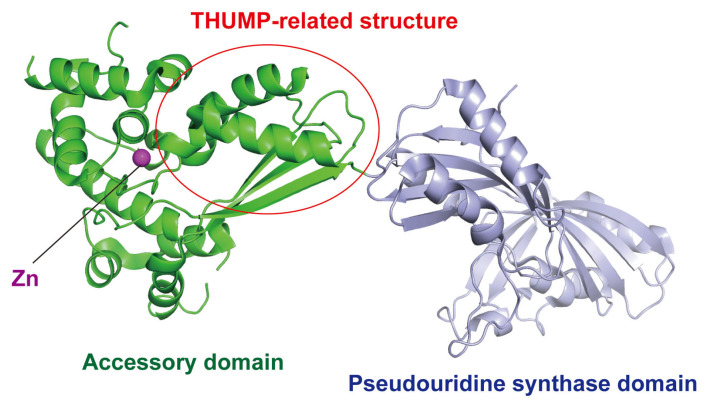
Structure of human PUS10 (PDB code, 2V9K) is represented by a cartoon model. *N*-terminal accessory and *C*-terminal pseudouridine synthase domains are colored in green and pale blue, respectively. The THUMP-related structure in the accessory domain is enclosed by a red circle. One Zn atom (magenta) is bound in the accessory domain.

In 2007, a crystal structure of human PUS10 was reported (Figure 8) and showed that the THUMP-related structure is contained in the *N*-terminal accessory domain [20]. When the CCA-terminus in tRNA is placed onto the THUMP-related structure, the modification sites (U54 and U55) have access to the catalytic pocket of the pseudouridine synthase domain [20]. However, human PUS10 can modify U54 in a tRNA transcript without a CCA terminus [30]. Because human PUS10 strongly recognizes the sequences of the aminoacyl-stem and T-arm [30], the recognition of the CCA terminus by the THUMP-related structure may be not important for pseudouridine formation. The accessory domain of human PUS10 is large compared to a typical THUMP domain. This large accessory domain was gained in the process of evolution of eukaryotic PUS10 [143]. Furthermore, tRNA recognition by human PUS10 in living cells is complicated. Human PUS10 is expressed in both the nucleus and cytoplasm [30]. Human nuclear PUS10 does not have the pseudouridine synthesis activity and inhibits the activity of TRUB1 [human tRNA (Ψ55) synthase] by binding to specific tRNAs in the nucleus [31]. In contrast, human cytoplasmic PUS10 can synthesize Ψ54 in tRNAs, which possess an AAAU sequence from position 57 to position 60 in the T-loop, in addition to Ψ55 [31]. Moreover, it has been reported that human PUS10 is involved in microRNA processing [152]. In this process, PUS10 directly binds to primary microRNA and the catalytic activity of PUS10 is not required [152]. Thus, PUS10 may act as an RNA binding subunit in microRNA processing.

Based on the crystal structure of human PUS10, a structural model of archaeal PUS10 was constructed and several amino acid residues, which are required for enzymatic activity and tRNA binding, were identified [21]. Another mutagenesis study revealed that the thumb-loop in the catalytic domain and *N*-terminal cysteine residues are important for the Ψ54 formation activity of *M. jannaschii* PUS10 [151].

## 5. Functions of Modified Nucleosides, Which Are Produced by THUMP-Related tRNA Modification Enzymes and Additional Information

In this section, the functions of modified nucleosides, which are produced by THUMP-related tRNA modification enzymes, are introduced. Furthermore, the relationships between the disorder of modification (or modification enzyme) and higher biological phenomena are explained.

### 5.1. s^4^U8 and s^4^U9

The s^4^U modification is observed at positions 8 and 9 in eubacterial and archaeal tRNAs [1,2,3]. The physiological roles of s^4^U have gradually been elucidated. The s^4^U modification in tRNA acts as an ultraviolet light (UV)-resistant factor [153]. Irradiation with near-UV causes crosslinking between s^4^U8 and C13 in tRNA [154]. Because ThiI requires the CCA terminus for the s^4^U modification, crosslinking by s^4^U occurs after the removal of the 3′-trailer sequence from precursor tRNA. This crosslinking of tRNA pauses protein synthesis and activates the DNA repair system [155,156]. Furthermore, crosslinking slows down the speed of TrmH-mediated Gm18 formation in tRNA [157]. Several archaea and bacteria live in environments in which sunlight does not reach (for example, deep sea and underground). However, these organisms also possess the s^4^U modification in tRNA [158], suggesting that the s^4^U modification functions beyond being a UV-resistant factor. As described in Section 3.1, the s^4^U8 modification contributes to the maintenance of the L-shaped tRNA structure. Furthermore, the s^4^U8 modification works as a tRNA quality control system in *Vibrio cholerae* in the stationary growth phase [159].

### 5.2. U8

Deamination from C8 to U8 performed by CDAT8 is one of the thermophile-specific tRNA modifications [17,62]. *M. kandleri* grows at high temperatures (more than 110 °C). Therefore, C8 in the tRNA genes may contribute to maintain the double-stranded DNA structure of the *M. kandleri* genome at high temperatures through an increase in the G-C content [17].

### 5.3. m^2^G6, m^2^_2_G6, m^2^G7, m^2^G10, m^2^_2_G10 and m^2^G67

The m^2^G modification does not disrupt formation of a Watson–Crick base pair with C, and the methyl group in m^2^G probably stabilizes the stem structure by hydrophobic interaction. The growth rate of a *S. cerevisiae trm11* gene deletion strain is comparable to that of the wild-type strain under laboratory conditions [23]. However, a *trm1*-*trm11* double-gene deletion strain shows an obvious growth defect [23]. Because Trm1 is the tRNA methyltransferase responsible for the formation of m^2^_2_G26 [102,103], the study [23] strongly suggests that the m^2^G10 modification works in co-ordination with other modification(s) in tRNA. In the case of *T. kodakarensis*, the *trm11* gene deletion strain cannot grow at high temperatures (95 °C) [113,160]. In *T. thermophilus*, the tRNA modification enzymes and modified nucleosides form a network in which modified nucleosides regulate the activities of other tRNA modification enzymes negatively and positively [62,63,161,162,163,164]. However, *trmN* gene deletion from the *T. thermophilus* genome does not have an effect on other modifications in tRNA [25]. This observation suggests that the m^2^G6 modification is a relatively late modification like dihydrouridine modification at positions 20 and 20a by DusA [165,166,167,168,169] in *T. thermophilus* tRNAs. In thermophiles, long and branched polyamines are produced [170,171] and have an effect on tRNA modifications [172,173]. In tRNA from the *T. thermophilus speB* or *speD1* gene deletion strain in which long and branched polyamines are not synthesized, the m^2^G6 modification in tRNA is increased [174]. Therefore, long and branched polyamines may negatively regulate m^2^G6 formation by TrmN in *T. thermophilus* cells. THUMD3 knockout HEK293T cell lines show decreased protein synthesis activity and an obviously slow growth rate [27]. Thus, human THUMPD3-TRMT112 is required for cell proliferation [27]. Furthermore, absence and presence of the m^2^G7 modification in tRNA^Trp^ are involved in the infection of avian retrovirus [175]. Moreover, although squid tRNA^Lys^ contains m^2^G67 [176], this modification is not explainable by the enzymatic activity of currently known eukaryotic tRNA methyltransferases.

### 5.4. ac^4^C12 and ac^4^C at Multiple Positions

Recent technologies, which can detect ac^4^C in RNAs, have shown that the ac^4^C modification is present in various RNAs beyond tRNA and rRNA [18,19]. As described in the Introduction, a THUMP-related protein, *S. cerevisiae* Tan1, was found to be an essential protein for ac^4^C12 modification in tRNA [14] but does not act in acetylation of 18S rRNA [14,16]. The *S. cerevisiae tan1* gene deletion strain shows a decrease in tRNA^Ser^ [14]. Furthermore, the *S. cerevisiae tan1* and *trm44* double mutant strain cannot grow at 33 °C [177]. Trm44 is a tRNA methyltransferase responsible for formation of Um44 in tRNA^Ser^ [177]. Thus, these studies show that ac^4^C12 contributes to stabilizing tRNA^Ser^ and works with other modifications such as Um44. Hypomodified tRNA^Ser^ is degraded by a rapid tRNA decay pathway, which competes with the elongation factor 1A [178]. *S. cerevisiae* Tan1 precursor-mRNA processing requires the conserved precursor-mRNA retention and splicing complex (RES complex; Bud13, Snu17 and Pml1 complex) [179]. Thereby, the RES complex controls ac^4^C12 modification in tRNA [179]. In the case of *T. kodkarensis*, ac^4^C modification by TkNAT10 occurs in various RNAs including tRNAs and is increased at high temperatures [18]. The acetylation by TkNAT10 is essential for survival of *T. kodakarensis* at high temperatures [18,160]. Loss of function of human THUMD1 causes a syndromic neurodevelopmental disorder [180]. The expression level of THUMD1 is increased in breast cancer cells [181]. Furthermore, THUMD1 overexpression enhanced breast cancer cells’ invasion and migration [181]. Moreover, although human NAT10 localizes mainly in nucleoli of normal tissues, it is redistributed to the membrane of colon cancer cells [182]. In addition, the expression level of NAT10 is increased in liver cancer [183].

### 5.5. ψ 54 and ψ 55

The modifications at positions 54 and 55 in tRNA stabilize the interaction between the T-arm and D-arm. Almost all tRNAs possess U modifications at position 54 (for example, m^5^U54, Ψ54, m^5^s^2^U54, m^1^ Ψ54, Um54, m^5^Um54, and s^2^Um54) and Ψ55 [3]. The Ψ54 modification is observed in tRNAs from archaea and some eukaryotes, and the Ψ55 modification is found in tRNAs from the three domains of life. Only higher eukaryotes and archaea possess PUS10 [28,29,184]. Consequently, eubacteria and yeast possess other enzymes. In the case of *E. coli*, TrmA [72] and TruB [73] catalyze the formation of m^5^U54 and Ψ55, respectively. In the case of yeast, m^5^U54 and Ψ55 are produced by Trm2 [185] and PUS4 [186], respectively. In archaea and higher eukaryotes, the Ψ55 modification in tRNA is synthesized by redundant systems. In archaea, archaeal Cbf5 (or archaeal Cbf5-Gar1 complex) and arcPUS10 can synthesize the Ψ55 modification [28,184]. In humans, nuclear TRUB1, mitochondrial TRUB2 and cytoplasmic PUS10 catalyze the formation of Ψ55 [31]. Consequently, cytoplasmic tRNAs are modified by TRUB1 or PUS10. Furthermore, it has been reported that PUS1 and PUS4 can synthesize the Ψ55 modification in *Cyanidioschyzon merolae* [187]. Although *C. merolae* does not possess PUS10, the redundant Ψ55 formation in tRNA is also observed in red algae. These facts suggest the importance of the Ψ55 modification. In *Haloferax volcanii* and *M. jannaschii*, the Ψ54 modification is further modified to m^1^ Ψ54 by TrmY [188,189]. Furthermore, in *Ignicoccus hospitalis*, the m^1^ Ψ54 modification is modified to m^1^s^4^ Ψ54 by TtuA and TtuB [190]. TtuA and TtuB are a sulfur-transfer complex responsible for the formation of s^2^U54 in tRNA [40,191]. The *PUS10* gene may be essential for survival of *H. volcanii* (the *PUS10* gene deletion mutant strain could not be obtained) [192]. In humans, mutations in *PUS10* gene are involved in Crohn’s disease and celiac disease (chronic intestinal inflammatory diseases) [193]. Human cytoplasmic PUS10 can synthesize Ψ54 in tRNAs, which possess an AAAU sequence from position 57 to position 60 in the T-loop, in addition to Ψ55 [30].

## 6. Perspective

In this review, I focus on the structures and functions of THUMP-related tRNA modification enzymes and the modified nucleosides they produce in tRNA. As described above, the studies of tRNA 4-thiouridine synthase, tRNA deaminase and tRNA methyltransferases have established the concept that the THUMP domain captures the 3′-end of RNA (the CCA-terminus of tRNA). The Tan1-Kre33 complex may have a similar recognition mechanism for substrate tRNA. However, TkTAN1-TkNAT10 modify multiple positions in tRNA. This phenomenon cannot be simply explained by our current knowledge. Furthermore, human PUS10 does not show the pseudouridine synthase activity in nucleus and is involved in processing of microRNA. Thus, functions and regulations of THUMP-related proteins in higher eukaryotes are complicated. Several THUMP-related proteins may be involved in the maturation of other RNAs beyond tRNA modifications. Moreover, there are many THUMP-related proteins for which the function is unknown. For example, the function of human THUMD2, which is predicted as a THUMP-related protein, is still unknown. Thus, further study will be necessary to clarify these issues.

## Figures and Tables

**Table 1:** Classification of tRNA modification enzymes with a THUMP domain.

Enzyme Type	Name	Modification and Position(s) in tRNA	References
4-thiouridine synthetase	*E. coli* and *Methanococcus maripaludis* ThiI	s^4^U8 and s^4^U9	[12,22]
deaminase	*Methanopyrus kandleri* CDAT8	U8	[17]
methyltransferase	*P. abyssi* Trm11 (arcTrm11)	m^2^G10 and m^2^_2_G10	[13]
	*S. cerevisiae* Trm11-Trm112 and *Archaeoglobus fulgidus* arcTrm11-arcTrm112	m^2^G10 (and m^2^_2_G10)	[23,24]
	*Thermus thermophilus* TrmN	m^2^G6	[25]
	*Methanocaldococcus jannaschii* Trm14	m^2^G6 and m^2^G67	[26]
	*Homo sapiens* THUMPD3-TRM112	m^2^G6 and m^2^G7	[27]
Partner protein of acetyltransferase	*S. cerevisiae* Tan1-Kre33	ac^4^C12	[16]
	*H. sapiens* THUMPD1-NAT10	ac^4^C12	[16]
	*Thermococcus kodakarensis* TkTHUMDP1-TkNAT10	ac^4^C (multiple positions)	[18]
Pseudouridine synthase	*Pyrococcus furiosus* and *M. jannaschii* Archaeal Pus10 (arcPus10)	Ψ54 and Ψ55	[28,29]
	*H. sapiens* PUS10	Ψ54 and Ψ55	[30,31]

**Table 2:** THUMP-related tRNA methyltransferases.

Enzyme Type	Organism	Subunit Composition	Modification(s)	Reference(s)
Trm11/arcTrm11/arcTrm11-arcTrm112/TRMT11-TRMT112	*S. cerevisiae*	Trm11-Trm112	m^2^G10	[23]
	*H. sapiens*	TRMT11-TRMT112	m^2^G10?	[111]
	*A. fulgidus*	arcTrm11-arcTrm112	m^2^G10 and m^2^_2_G10	[24]
	*H. volcanii*	arcTrm11-arcTrm112	m^2^G10 and m^2^_2_G10	[112]
	*P. abyssi*	arcTrm11	m^2^G10 and m^2^_2_G10	[13]
	*T. kodakarensis*	arcTrm11	m^2^G10 and m^2^_2_G10	[34,113]
TrmN/Trm14/THUMPD3-TRMT112	*T. thermophilus*	TrmN	m^2^G6	[25]
	*M. jannaschii*	Trm14	m^2^G6 and m^2^G67?	[26]
	*T. kodakarensis*	Trm14	m^2^G6 and m^2^G67?	[113]
	*H. sapiens*	THUMPD3-TRMT112	m^2^G6 and m^2^G7	[27]
